# Anatomical Variations That Can Lead to Spine Surgery at The Wrong Level: Part II Thoracic Spine

**DOI:** 10.7759/cureus.8684

**Published:** 2020-06-18

**Authors:** Manan Shah, Dia R Halalmeh, Aubin Sandio, R. Shane Tubbs, Marc D Moisi

**Affiliations:** 1 Neurosurgery, Wayne State University, Detroit Medical Center, Detroit, USA; 2 Neurosurgery, Detroit Medical Center, Detroit, USA; 3 Neurosurgery and Structural & Cellular Biology, Tulane University School of Medicine, New Orleans, USA; 4 Anatomical Sciences, St. George's University, St. George's, GRD; 5 Neurosurgery and Ochsner Neuroscience Institute, Ochsner Health System, New Orleans, USA

**Keywords:** surgery at the wrong level, thoracic spine, anatomical variations, spine, sentinel events, thoracic, congenital

## Abstract

Spine surgery at the wrong level is a detrimental ordeal for both surgeon and patient, and it falls under the wrong-site surgery sentinel events reporting system. While there are several methods designed to limit the incidence of these events, they continue to occur and can result in significant morbidity for the patient and malpractice lawsuits for the surgeon. In thoracic spine, numerous risk factors influence the development of this misadventure. These include anatomical variations such as transitional vertebrae, rib variants, hemivertebra, and block/fused vertebrae as well as patient characteristics, such as tumors, infections, previous thoracic spine surgery, obesity, and osteoporosis. An extensive literature search of the PubMed database up to 2019 was completed on each of the anatomical entities and their influence on developing thoracic spine surgery at the wrong level, taking into consideration patient’s individual factors. A reliable protocol and effective techniques were described to prevent this error. In addition, the surgeon should collaborate with radiologists, particularly in challenging cases. A thorough understanding of the surgical anatomy and its variants coupled with patients characteristic is crucial for maximal patient benefit and avoidance of thoracic spine surgery at the wrong level.

## Introduction and background

Spine surgery at the wrong level has tremendous clinical and emotional implications for the patient and surgeon. It can lead to additional procedures and risks, damage the doctor-patient relationship, and usually associated with legal actions [1]. It is part of the wider field of wrong-site surgery [1,2]. According to the Joint Commission on Accreditation of Healthcare Organization (JCAHO), wrong-site surgery was the most common sentinel event (13%) in 2008 [1]. The prevalence of spine surgery at the wrong site reported in the literature ranges from 0.09 to 4.5 per 10,000 surgeries performed [3]. Out of 415 spine surgeons, 207 (approximately 50%) reported that they had performed at least one such surgery in their career [3]. In a questionnaire study encompassing 1,300,000 spine surgeries, 418 procedures had been performed at the wrong level, with 8% performed on the thoracic region, whereas 71% were performed on the lumbar and 21% on the cervical regions [1]. However, due to huge diversity of the published data about incidence of spinal surgery at the wrong level, a clear-cut incidence rate has not been established [1]. Unfortunately, because of its complex anatomy and higher number of vertebrae, surgery at the wrong level in the thoracic spine continues to occur.

In this article, we describe several of the thoracic spine anatomical variations such as thoracolumbar transitional vertebrae, rib variants, hemivertebra, and block/fused vertebrae, and patient characteristics including tumors, infection, previous thoracic surgery, obesity, and osteoporosis that might result in greater risk of surgery at the wrong level. Therefore, it is critical to understand these thoracic spine anomalies to decrease the likelihood of such incorrect surgery. Moreover, we explain important techniques and preventive strategies that can be utilized to limit wrong-level thoracic spine surgery.

## Review

Material and methods

Materials for this review were searched on the PubMed database using the following key words “wrong level surgery, thoracolumbar transitional vertebrae, thoracic rib abnormalities, thoracic hemivertebra, thoracic congenital fused vertebrae, thoracic spine anomalies, obesity and spine surgery, osteoporosis and spine surgery” individually or in combination, up to 1981. The search was performed over a period of three months, between October 2018 and December 2018. Original articles regarding the association between thoracic spine anatomical variations and development of wrong-level thoracic spine surgery were included. In addition, few articles were extracted based on information provided by the senior authors. Corresponding publications on potential risk factors such as unusual patient characteristics were also included. Articles that reported none of the previously mentioned correlations, and duplications among the database were excluded. Then, the articles were filtered to include full text articles and English-only publications. Finally, they were reviewed and those most representative were selected.

Results and discussion

Out of over 6,000 articles found using the initial search terms noted in the methods section, and after going through the abstracts of related articles and further review, there were 23 eligible articles for this literature review. We summarize the potential thoracic anatomical variations and patients characteristics that can lead to thoracic spine surgery at the wrong level as follows:

Transitional Vertebrae

Thoracolumbar transitional vertebrae are those in which features of the thoracic and lumbar segments at the thoracolumbar junction are retained in part [4-6]. Wigh et al. defined a thoracolumbar vertebra as a segment with a short rib, <3.8 cm in length or with an accessory ossification center [6]. Carrino et al. defined it as a vertebra with a rib on one side and a transverse process on the contralateral side [7]. Park et al. identified five types of thoracolumbar transitional vertebrae: Type I, IIA, IIB, III, and IV [4]. Type I includes paired ribs with more than one short rib; type IIA includes a 3.8 cm or longer rib on one side and an accessory ossification center or transverse process on the other; type IIB includes a short rib on one side and an accessory ossification center or transverse process on the other; type III includes a unilateral or bilateral mixed type rib, and type IV includes a unilateral or bilateral accessory ossification center [4]. The frequency of these transitional vertebrae was 12.6% in their study. It is important to understand transitional vertebrae’s morphology to reduce errors in spinal enumeration and prevent surgery at the wrong level.

Rib Variants 

**Figure 1:**
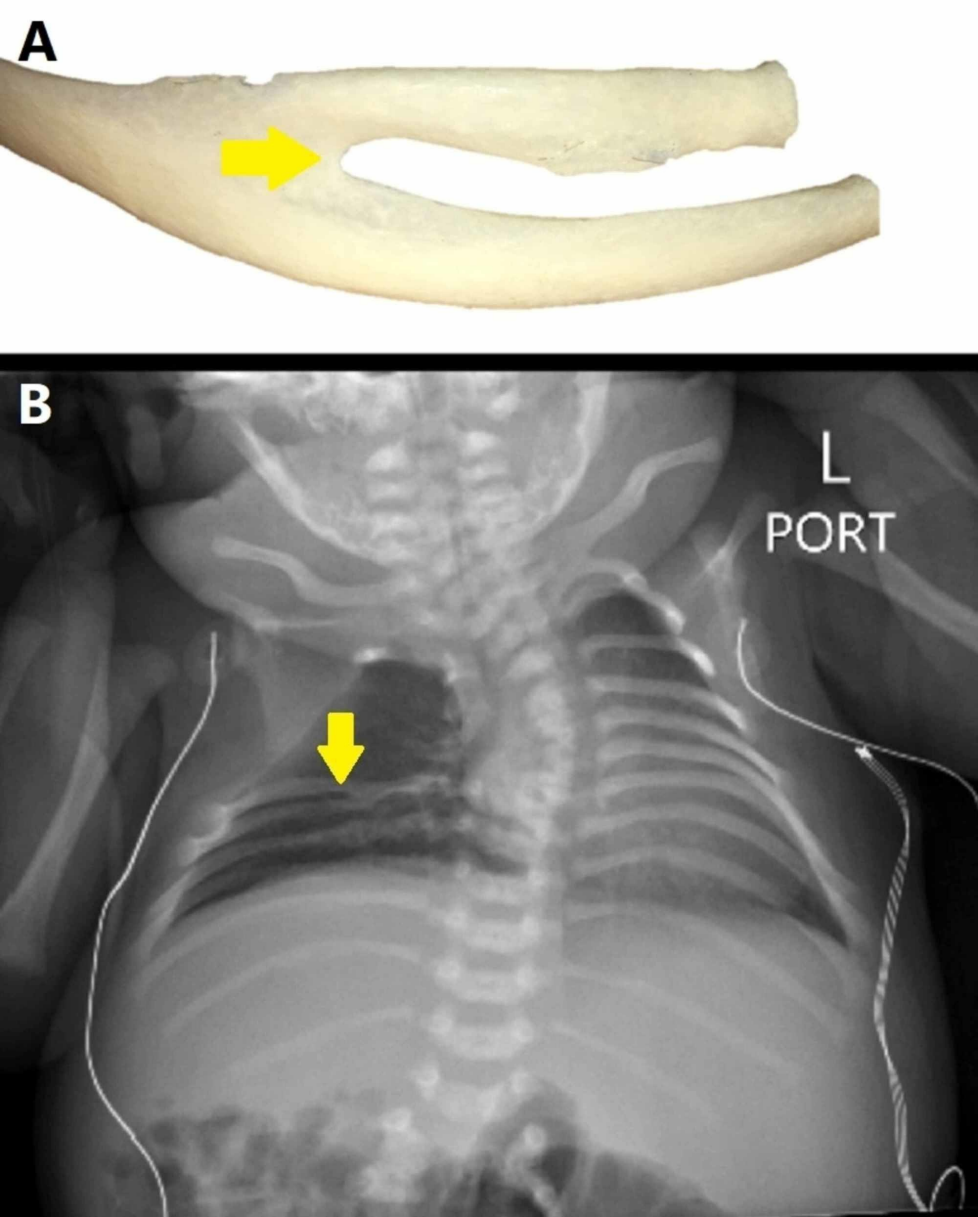
Bifid rib (A) Closer lateral view of a right bifid rib. Note the bifurcation before attaching to the sternum (yellow arrow). (B) Radiograph of a child with with right-sided fork-type bifid ribs with mild scoliosis (yellow arrow). Bifid rib, which usually has no associated symptomatology, is a bifurcation of the anterior portion of the rib before fusion with the sternum. There is also bifurcation of the associated neurovascular bundle [8].

The ribs in the thoracic spine have various numerical and structural abnormalities, all of which can increase the risk of surgery at the wrong level. One study showed that the incidence of congenital rib abnormalities in the general population was 1.4% [8], while the incidence of a bifid type rib was 0.6%, fusion type rib was 0.3%, and hypoplastic and rudimentary rib was 0.2% [8]. The bifid type rib is a variant in which the rib’s sternal end is cleaved in two, and usually is unilateral (Figure 1). It is more common in females, occurs more frequently on the right side, and is seen most commonly in the 3rd and 4th ribs [8,9]. Further, fusion of two or more ribs can be seen in certain individuals.

**Figure 2:**
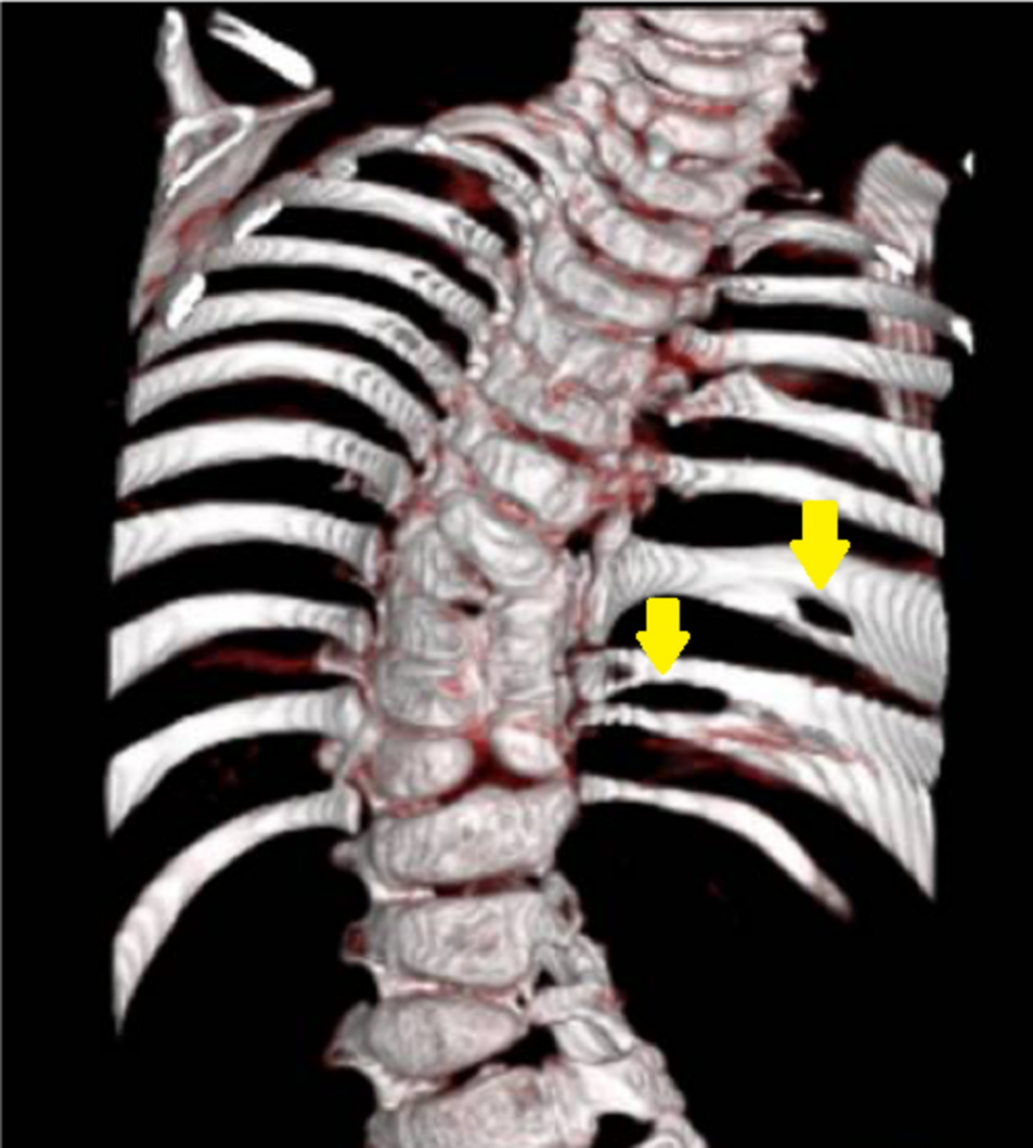
Hole-type bifid rib Hole-type bifid ribs on the left side nearer to the vertebral column (arrows) with narrowing of the lower intercostal space between both bifids

In Davran et al.’s study, the most common form was fusion of the 1st and 2nd ribs [8]. In addition, Davran et al discovered that the most common type was the hole type [8]. Visually, this type of bifid rib can present with a narrower lower intercostal space (Figure 2). Hypoplastic, or short, ribs are one of the least common of the rib’s structural abnormalities. A rib is considered hypoplastic if its lateral margin is more than 4 mm medial to a tangent drawn between the adjacent ribs’ lateral margins [8]. Hypoplasia or aplasia of the rib can be seen together with Down’s syndrome and Poland’s syndrome [8,10,11]. In Davran et al.’s study, a rudimentary 12th rib also was found in 9.62% of cases [8]. Intraoperatively, the ribs often are used to identify the correct level in the thoracic spine. Therefore, preoperative imaging is essential to identify these rib variants, and thereby necessary to prevent errors in level identification during surgery.

Hemivertebra

**Figure 3:**
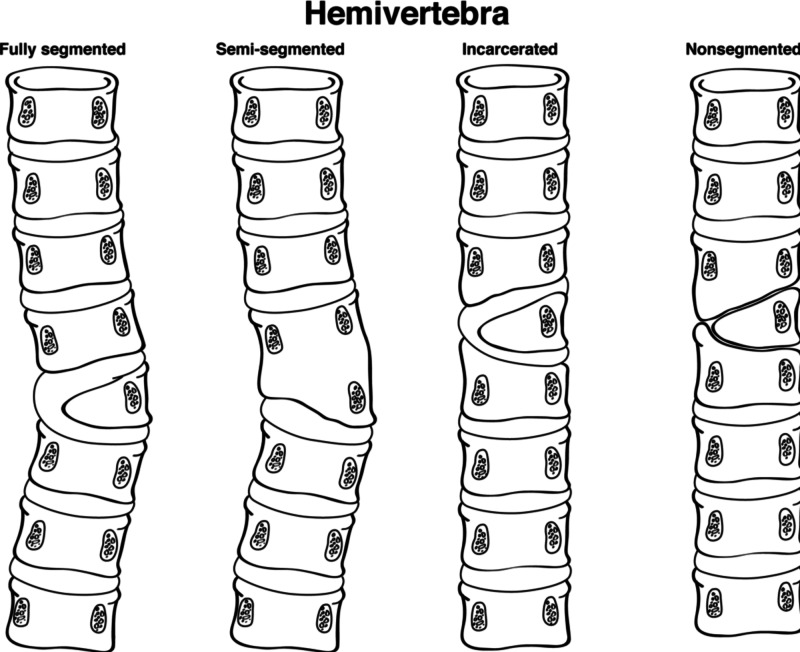
Classification of hemivertebrae The formation of a hemivertebrae is due to failure of an ossification leading to anomalies in the vertebrae which can result in kyphosis, lordosis. The four types of Hemivertebrae: fully segmented, semi-segmented, incarcerated, and nonsegmented (left). Adapted from 'Hemivertebrae: a comprehensive review of embryology, imaging, classification, and management', by J Johal, M Loukas, C Fisahn, JR Chapman, RJ Oskouian, RS Tubbs, 2016, Child's Nervous System [14].

Hemivertebra, in which only half of the vertebral body develops, is another variant that can occur in the thoracic spine. The incidence is approximately 5-10 per 10,000 births and is more common in girls [12]. It is a congenital variant that develops when one of two chondrification centers fails to form [12]. It is most commonly found in the midthoracic spine, particularly at the T8 level [13]. There are four distinct types: Incarcerated, nonincarcerated, segmented, and unsegmented (Figure 3) [13]. Incarcerated hemivertebrae are those in which the vertebral bodies above and below the abnormal segment accommodate the hemivertebrae, while nonincarcerated refers to the failure of accommodation, which results usually in spinal curvature. Segmented, or free, hemivertebrae have a normal disk above and below the defective body and are more likely to lead to progressive curvature, while unsegmented hemivertebrae are fused with the vertebral body above and below [13]. Hemivertebrae can lead to angulation of the thoracic spine, resulting in kyphosis, lordosis, or scoliosis. This anomaly can make it difficult to identify the correct thoracic level during surgery, and careful examination of radiographs should be exercised. Indeed, some evidence has indicated that it is beneficial to obtain a pre-operative CT when a hemivertebra is identified. After identification, it can be used as a landmark during thoracic spine surgery.

Block Vertebra

Block vertebra is a congenital anomaly that is most common in the cervical and lumbar spine, but can occur in the thoracic spine as well, and is attributable to the vertebral column’s improper segmentation [15]. Fusion of adjacent vertebrae occurs through their intervertebral disc, and can lead to an abnormal angle in the spine [15]. This anomaly can cause improper numerical labeling of vertebra and potentially surgery at the wrong level. However, once this variation is identified, it can be used as a landmark to confirm other levels during surgery.

Tumors and Infection

Spinal tumors, particularly spinal metastatic lesions, can alter the spine’s anatomy. Further, lytic lesions sometimes make it difficult to identify the thoracic vertebrae with X-ray imaging. Infectious lesions to the spine, such as osteomyelitis/diskitis, also can distort the normal bony anatomy. Chronic infections can destroy the intervertebral disc and fuse the vertebral bodies, and therefore, careful preoperative review of imaging is key to prevent surgery at the wrong level. 

Previous Spine Surgery

Previous thoracic surgery alters the normal anatomy and makes it more difficult to localize the correct level for a reoperation, and bony defects complicate intraoperative identification of the proper level. In addition, scar tissue also makes visual identification more challenging. In patients with prior instrumentation, X-ray imaging must be reviewed more carefully to identify the anatomy. Ideally, the surgeon should analyze the surgical procedure performed previously to avoid surgery at the wrong level. 

Obesity and Osteoporosis

Obesity and osteoporosis are comorbid conditions that also can increase the risk of performing thoracic spine surgery at the wrong level. 34.9% of U.S. adults, or 78.6 million people, are estimated to be obese. Its incidence is rising in the general population, and spine surgeons are operating more and more on these individuals [16]. The number of spine operations in elderly patients also is increasing, as is the incidence of osteoporosis in spine-surgery patients [17]. Both large body habitus and decreased bone density can cause inadequate radiological exposure [1]. The shoulders in patients with a large body habitus also can prevent high quality intraoperative X-rays of the upper thoracic spine. All of these patient characteristics must be recognized before surgery to avoid surgery at the wrong level.

Strategies to Prevent Thoracic Spine Surgery at the Wrong Level

All of the thoracic spine anatomical variations and patient characteristics described above can contribute to surgery at the wrong level. Accordingly, it is imperative for the surgeon to formulate a reliable routine. Preventing surgery at the wrong level begins in the clinic where the surgeon must ensure that the patient is seen and agrees with the correct side and level(s) [18]. The preoperative imaging, X-ray, CT, and/or MRI, should be analyzed to identify any anatomical variations. Radiographs of the entire spine allow radiologists to count from C2 inferiorly. In addition, comorbid conditions such as obesity and osteoporosis should be identified in patients undergoing surgery. In those patients where difficulty in counting levels is anticipated, interventional radiology can place fiducial markers preoperatively [18,19-21]. Advanced fiducial markers, including percutaneous fiducial screws and skin adhesive radio-opaque grid lines, also have been shown to improve the accuracy of spinal level localization [19-21]. Another preoperative technique described is placing polymethylmethacrylate into the vertebral body percutaneously using standard vertebroplasty [19,22].

Careful examination of preoperative images in the OR should always be performed before initiating the surgery. High quality intra-operative X-rays are essential so that the level(s) of interest can be counted clearly [18]. The intraoperative X-ray imaging should be done multiple times for verification in cases with confusing anatomy. A radiologist can assist with obtaining good quality X-rays and identification of the target levels intraoperatively. A spinal needle can be inserted systematically between the spinous processes and, combined with a lateral X-ray, can help the surgeon navigate to the correct interspace. An oblique modification of the cross table lateral X-ray has been shown to improve visualization of the thoracic levels by removing the shoulder and the majority of the ribs from the field of view [19,23]. Certain other methods to identify the proper level include intraoperative CT, spinal neuronavigation, and transligamentous ultrasound [1,3]. When instrumentation is used, a postoperative X-ray also is recommended to verify proper placement of instrumentation and the levels, preferably before the incision is closed [18]. These strategies can help decrease the risk of surgery at the wrong level.

## Conclusions

Thoracic spine surgery at the wrong level is a devastating experience for surgeons and their patients. It can lead to unnecessary surgeries and additional risks for the patient and damage the relationship between the surgeon and the patient. Numerous factors can contribute to surgery at the wrong level in the thoracic spine; however, in this literature review, we have focused on several thoracic spine anatomical variations and patient characteristics that can lead to this surgical error. Identifying these variations and analyzing imaging preoperatively and intraoperatively is crucial to avoid this pitfall. Often, working with radiologists can clarify challenging anatomy, and it is imperative for the surgeon to use the previously mentioned techniques to avert performing thoracic spine surgery at the wrong level.
